# Cardiac action potential generation mechanisms via an intramembrane photoswitch. A simulation study

**DOI:** 10.1016/j.bpj.2025.04.029

**Published:** 2025-05-03

**Authors:** Ludovica Cestariolo, Chiara Florindi, Chiara Bertarelli, Antonio Zaza, Guglielmo Lanzani, Francesco Lodola, Jose F. Rodriguez Matas

**Affiliations:** 1 Department of Biotechnology and Biosciences, Università degli Studi di Milano-Bicocca, Milan, Italy; 2 Department of Chemistry, Materials and Chemical Engineering “Giulio Natta,” Politecnico di Milano, Milan, Italy; 3 Center for Nano Science and Technology, Istituto Italiano di Tecnologia, Milan, Italy; 4 Department of Physics, Politecnico di Milano, Milan, Italy

## Abstract

Optical stimulation is emerging as a promising alternative to conventional methods for both research and therapeutic purposes due to its advantages, such as reduced energy consumption, minimal invasiveness, and exceptional spatial and temporal precision. Recently, we introduced Ziapin2, a novel light-sensitive azobenzene compound, as a tool to modulate cardiac cell excitability and contractility. The molecule proved to be effective in precisely regulating the excitation-contraction coupling process in both hiPS-derived cardiomyocytes and adult mouse ventricular myocytes (AMVMs). Experimental evidence suggests that stretch-activated channels (SACs) contribute to light-driven action potential (AP) generation, but the exact way this takes place remains unknown due to system complexity and lack of specific SAC blockers. Here, we aim to clarify the role of SACs and photostimulation mechanism by exploiting a computational model of murine AP that incorporates: 1) the variation in membrane capacitance resulting from the *trans*-*cis* isomerization of the molecule in response to light stimulation and 2) SACs activated by membrane tension due to the thickness variation induced by Ziapin2. Our numerical model accurately reproduces cell capacitance and membrane potential alterations induced by Ziapin2 photoisomerization. In addition, it elucidates the behavior observed experimentally in vitro in AMVMs, highlighting the pivotal role of calcium (Ca^2+^)-selective SACs in AP generation. The proposed model is thus a valid tool for cell behavior prediction in future experiments.

## Significance

The modulation of membrane capacitance through photomechano-transducers has been effectively demonstrated in both model membranes and living cells. This in silico study sheds light on the mechanisms governing the light-driven regulation of cardiac bioelectricity facilitated by Ziapin2, a recently synthesized intramembrane photoswitch capable of modulating the excitation-contraction coupling process in murine- and human-derived cardiomyocytes. By correlating mechanical perturbations with electrical responses, we further validate and expand our previous experimental observations, emphasizing the pivotal role of Ca^2+^-selective stretch-activated channels in light-induced action potential generation. These findings enhance our understanding of the biophysical mechanism at play and pave the way for developing novel strategies for the precise and noninvasive control of cardiac cellular excitability.

## Introduction

In the cardiac field, the use of light provides a clean, precise, and noninvasive method for controlling and influencing a wide range of biological processes in both in vitro and in vivo environments without causing damage (1). Compared with electrically driven stimulation methods, optical techniques offer superior spatiotemporal resolution, reduced energy consumption, minimal invasiveness, and faster response times, unlocking new opportunities in areas such as neuroscience, chemical biology, and photopharmacology. However, standard biotargets, such as eukaryotic cells, are typically unresponsive to light due to their low sensitivity to it. For this reason, researchers have developed various strategies to impart photosensitivity to cells, including the use of optogenetic tools (2,3), photosensitive nanomaterials (4,5,6,7), organic semiconductors (8,9,10), and molecular photoswitches (11,12). In particular, these molecular optical switches further expand photostimulation possibilities by enabling reversible modulation of cell sensitivity through precise control of the spatial arrangement of molecules (13,14,15).

Within this context, we proposed Ziapin2, a light-sensitive amphiphilic azobenzene compound that acts as a photoresponsive agent, modulating cell electrical properties primarily through structural changes in the membrane (16,17,18,19,20,21). Notably, Ziapin2 has been shown to optically trigger action potentials (APs) in excitable cells (16,22,23) and, when introduced into cardiac cultures, the resulting electrical modulation is associated with changes in calcium (Ca^2+^) dynamics and an increased contraction rate, suggesting its potential to regulate the entire excitation-contraction coupling process (22,23,24).

Recently, we conducted a detailed electrophysiological analysis of the light-induced modulation driven by Ziapin2 in adult mouse ventricular myocytes (AMVMs) (23). This study explored the molecular mechanisms at play, assessing the biophysical processes that initiate AP generation and investigating the role of channels whose opening probability is closely tied to membrane changes induced by Ziapin2. Given the observed perturbations in membrane thickness, the stretch-activated channels (SACs) (25) could alter their open probability when Ziapin2 is present, contributing to light-induced AP generation. Indeed, we provided experimental evidence indicating that SACs act as key mediators in translating the local membrane structure deformation induced by Ziapin2 into cellular physiological responses (23). While Ca^2+^-permeable SACs seem to play a critical role, the absence of specific pharmacological blockers and the complexity of the biological framework necessitate further investigation to precisely determine the ion currents involved and their temporal dynamics.

This study aims to enhance our understanding of the role of SACs by proposing an advanced computational model of murine AP (26), incorporating: 1) variations in membrane capacitance resulting from the *trans-cis* isomerization of the molecule in response to light stimulation and 2) the formulation of nonselective cation SACs (SAC_ns_) (27), which primarily conduct sodium (Na^+^) and potassium (K^+^) (and, to a lesser extent, Ca^2+^), as well as selective channels, including K^+^-selective (SAC_K_) and Ca^2+^-selective SACs (SAC_Ca_) (28).

## Materials and methods

To investigate the mechanisms behind the functioning of Ziapin2, a numerical murine model was developed using the Li et al. model (26) from 2009 as a starting point. The model was modified to incorporate time-dependent variations in membrane capacitance and, by the introduction of SACs, to account for the main observed effects associated with Ziapin2.

The experimental data used to parameterize the model were taken from Florindi et al. (23). Therefore, for detailed experimental procedures, please refer to this study. For convenience, a summary of the main methods used is provided here.

(1) AMVMs were freshly isolated by enzymatic digestion from 3- to 4-month-old mice.
(2) AMVMs were incubated with Ziapin2 as previously described. After 7 min, the cells were perfused with a fresh extracellular solution to remove the molecule that had not been internalized.
(3) Electrophysiological recordings were performed at 36°C using a patch-clamp technique. Both voltage and current-clamp (I = 0) experiments were performed by the ruptured-patch version of the whole-cell mode.
(4) For capacitance measurements, a dual-sinusoidal voltage-clamp protocol was applied in a whole-cell configuration. The resulting current response was recorded, and membrane capacitance and resistance were extracted.
(5) Illumination during the experiments was provided by a Thorlabs (Newton, NJ, USA) collimated light-emitting diode coupled to the fluorescence port of a Nikon (Tokyo, Japan) Eclipse TE200 inverted microscope. The external light source had a peak emission wavelength of 470 nm, selected to match the absorption spectrum of the molecule.

To facilitate the comparison between experimental data obtained from different cells, the traces have been offset to start from a resting potential of 0 mV. Consequently, the numerical model was also offset when compared with the experimental data. In all other cases, the transmembrane potential values are reported without any offset.

### Membrane capacitance variations

According to experimental results, upon partitioning into the cardiomyocyte sarcolemma, Ziapin2 undergoes dimerization in dark conditions, anchoring to opposite leaflets of the membrane and leading to local thinning, which increases membrane capacitance. In previous cell experiments (23), exposure to the molecule in dark conditions did not result in discernible differences. This was reproduced also in numerical simulations (e.g., by increasing $C_{m}$). As shown in Fig. S1, the $C_{m}$ increment only slightly delayed final repolarization.

**Figure 1:**
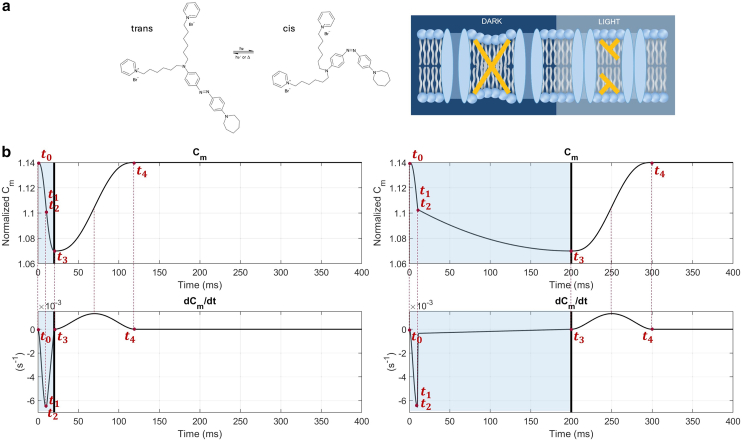
Effects of Ziapin2 partitioning in the cell membrane. (*a*) Ziapin2 molecular structure and its light-induced isomerization. In the *trans* conformation, Ziapin2 molecules attach to opposite leaflets of the membrane, forming dimers. This leads to local membrane thinning, which increases membrane capacitance (*dark*). When exposed to millisecond pulses of visible light (*λ* = 470 nm), Ziapin2 undergoes isomerization into its *cis* form. In this state, the hydrophobic tails of opposing molecules are too distant to dimerize, resulting in a rapid capacitance drop due to membrane relaxation (*light*). (*b*) $C_{m}$ numerical variations over time and its relative derivative for two different light stimulation durations (20 ms in the *left panel*, 200 ms in the *right panel*). The red dots represent the instants $t_{1}$, $t_{2}$, $t_{3}$, and $t_{4}$, which can be adjusted in the code to simulate different stimulation patterns. It should be noted that $t_{1}$ and $t_{2}$ are nearly coincident, as segment two is designed to be very short, ensuring the continuity of derivatives. Photoexcitation is represented by the blue shaded area, and the black line indicates the end of the light stimulation.

When exposed to millisecond pulses of visible light (λ = 470 nm), Ziapin2 undergoes *trans-cis* isomerization, disrupting the dimers as the hydrophobic tails of opposing molecules become too distant to interact. This allows a partial restoration of the initial thickness of the membrane, resulting in a consequent decrease in the value of the capacitance. The action of Ziapin2 under dark and light conditions is schematically shown in Fig. 1 *a*.

A modified parallel conductor model for the cell was adopted to account for the cascade of effects consequent to the time-dependent change in capacitance. The membrane is represented as a capacitor with time-dependent capacitance in parallel with nonlinear resistances and batteries, which represent the ionic channels and current sources used to model pumps and exchangers. Consequently, the electrophysiological behavior of the cell can be described by:

(1) $$C_{m}\frac{dV_{m}}{dt}=-J_{ion}-V_{m}\frac{dC_{m}}{dt}$$

where $C_{m}$ is the time-dependent specific cell membrane capacitance, $V_{m}$ is the transmembrane voltage, t is time, and $J_{ion}$ is the sum of the ionic currents.

$C_{m}$ values in the model are based on experimental data (23), considering the value for the control case (i.e., no Ziapin2) as unit and scaling proportionally for the other two conditions, as reported in Table 1.

**Table 1:** $C_{m}$ Variation due to Ziapin2 partitioning in the cell membrane

Condition	Experimental (pF)	Numerical (–)
Control	245.7	1
Ziapin2, dark	281.7	1.14
Ziapin2, light	263.6	1.07

$C_{m}$ values evaluated experimentally (23) and their corresponding numerical values.

The time dependence of $C_{m}$ and, consequently its derivative, have been divided into four segments, reproducing the expected physical phenomena. The duration of each step is adjusted according to the light stimulation duration (see Fig. 1 *b*).

The $t_{0}$-$t_{1}$ phase represents the rapid change in the molecule configuration due to the light stimulation, the $t_{1}$-$t_{3}$ phase represents the recruiting phase in which more molecules engage the configuration change, whereas the final $t_{3}$-$t_{4}$ phase represents the recovery change after the light stimulation is removed. The definition of the time-dependent curve is such that continuity in the time derivative of $C_{m}$ is guaranteed. The list of the detailed equations for all four segments is reported in the supporting material.

### SAC formulation

**Figure 2:**
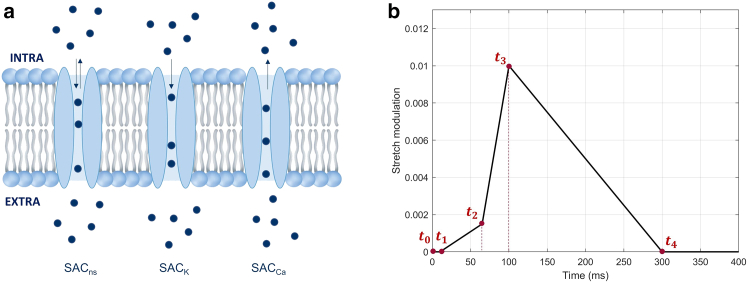
SACs functioning in the model. (*a*) SACs considered in the model. Non-selective channels (SAC_ns_), K^+^-selective channels (SAC_K_), and Ca^2+^-selective channels (SAC_Ca_). (*b*) Representative strain (λ−1) variations over time used in the model. The red dots represent the instants $t_{1}$, $t_{2}$, $t_{3}$, and $t_{4}$, which can be adjusted in the code to simulate different stretch patterns. It should be noted that the magnitude of the stretch can also be adjusted to reproduce different cases.

Experiments show that changes in membrane capacitance activate SACs (23). SACs in the sarcolemma have been reported in isolated myocytes, multicellular preparations, and the whole heart. Computational models for SACs have been proposed by Niederer and Smith (27), who focused on nonselective SACs (SAC_ns_), and K^+^-selective SACs (SAC_K_), and by Buonocunto et al. (28), who also considered Ca^2+^-selective SACs (SAC_Ca_). In this work, we implemented the same formulations. Specifically, the current through SAC_ns_, $I_{ns}$, is divided into Na^+^ (inward current) and K^+^ (outward current) components, as proposed by Niederer and Smith (27). While SAC_ns_ contribute to inward and outward currents, SAC_K_ contribute to an outward current, and SAC_Ca_ contribute to an inward current. The three SACs considered in the model are shown in Fig. 2 *a*.

The relative conductance of Na^+^ and K^+^ through SAC_ns_ ($r=g_{Na}/g_{K}$) can be estimated using the reversal potential of $I_{ns}$ ($E_{ns}$) and the reversal potential of Na^+^ and K^+^ using the following (Eq. 2):

(2) $$r=\frac{E_{ns}-E_{K}}{E_{Na}-E_{ns}}$$

The resulting SACns current (Eq. 3) is defined by:

(3) $$I_{ns}=I_{ns,Na}+I_{ns,K}$$

where $I_{ns,Na}$ and $I_{ns,K}$ are given by Eqs. 4 and 5, respectively:

(4) $$I_{ns,Na}=rg_{ns}\gamma_{SL,ns}\left(V_{m}-E_{Na}\right)$$

(5) $$I_{ns,K}=g_{ns}\gamma_{SL,ns}\left(V_{m}-E_{K}\right)$$

with

(6) $$\gamma_{SL,ns}=\beta_{ns}\left(\lambda -1\right)$$

The stretch-dependent K^+^ specific current through SAC_K_ (Eq. 7) is defined by:

(7) $$I_{Ko}=g_{Ko}\frac{\gamma_{SL,Ko}}{1+e^{-\frac{10+V_{m}}{45}}}\left(V_{m}-E_{K}\right)$$

with

(8) $$\gamma_{SL,Ko}=\beta_{Ko}\left(\lambda -1\right)+0.7$$

where λ is the local stretch of the membrane, $g_{ns}$ and $g_{Ko}$ the maximum conductances, and $\beta_{ns}$ and $\beta_{Ko}$ the strain coefficients for nonspecific SACs and K^+^-specific SACs, respectively.

Lastly, following the work of Buonocunto et al. (28), the stretch-activated Ca^2+^-specific current was defined as in Eq. 9:

(9) $$I_{CaP}=g_{CaP}\left(\lambda -1\right)z_{Ca}^{2}\frac{V_{m}F^{2}}{RT}\times \frac{\gamma_{Cai}{\left[{Ca}^{2+}\right]}_{i}e^{\left(\frac{z_{Ca}V_{m}F}{RT}\right)}-\gamma_{Cao}{\left[{Ca}^{2+}\right]}_{o}}{e^{\left(\frac{z_{Ca}V_{m}F}{RT}\right)}-1}$$

where $g_{CaP}$ is the channel conductance, $z_{Ca}$ is the charge of a Ca^2+^ ion, and F, R, and T are conventional thermodynamic constants. $\gamma_{Cai}$ and $\gamma_{Cao}$ are the intracellular and extracellular ionic activity coefficients, and ${\left[{Ca}^{2+}\right]}_{i}$ and ${\left[{Ca}^{2+}\right]}_{o}$ are the intracellular and extracellular Ca^2+^ concentrations, respectively. The selective K^+^ channel conductance $g_{Ko}$ is based on the work of Buonocunto et al. (28), where it was estimated based on experimental patch-clamp data from Li et al. (29). The conductance $g_{ns}$ from Buonocunto et al. (28), calibrated to experimental data from isolated human atrial cardiomyocytes, was adjusted with a scaling factor of 35 based on experimental expression data from mice and human atria samples (30), which showed an ∼35-fold larger mRNA expression in mice. Finally, the channel conductance $g_{CaP}$ was adjusted with a scaling factor of 0.9 according to experimental observations by Kim (31), which showed that its numerical value was very close to that of $g_{Ko}$. The parameters used in the model are provided in Table 2.

**Table 2:** SACs parameter

Parameter		Value
Maximal conductance (mS/μF)	$g_{ns}$	3.6121 × 10^−4^
	$g_{Ko}$	4.4873 × 10^−4^
	$g_{CaP}$	4.0386 × 10^−4^
Stretch coefficient (–)	$\beta_{ns}$	0.2203
	$\beta_{Ko}$	0.8742
Relative conductance of Na^+^/K^+^ (−)	r	1.411

List of parameter values used in the model.

During light/dark stimulation, the membrane thickness changes due to the conformational changes of the Ziapin2. Since we assume the cell membrane to be incompressible, changes in its thickness imply changes in membrane surface area, leading to local stretching that triggers activation of SACs. Considering the inverse proportional relationship between cell membrane capacitance and cell membrane thickness, together with an in-plane isotropic behavior of the cell membrane, implies an average membrane stretch in the order of 3% for a 7% change in capacitance between light and dark conditions (indicated in Table 1). During light stimulation, an increment in the membrane thickness due to *trans-cis* isomerization induces a general relaxation of the cell membrane, producing a relatively low SACs activation. On the contrary, when the light stimulation is interrupted and the molecule returns to its *trans* configuration, the membrane thickness decreases, causing a general stretch of the membrane that triggers activation of SACs. In addition, since the returning to *trans* configuration occurs much more slowly than *trans-cis* isomerization, membrane stretching is assumed not to occur immediately after the light stimulation is interrupted. Further, we also consider that the stretching of the membrane is not uniform and that the local stretch sensed by the SAC could be different from the average membrane stretch.

Based on these considerations, the local stretch applied to the SACs was divided into four phases, as shown in Fig. 2 *b*. In the first 10 ms of light stimulation ($t_{1}$), the membrane relaxes, SACs are inactivated, and, thus, no stretch is applied to them. The only contribution during this phase comes from capacitance variations. Therefore, $t_{1}$ was set to 10 ms for all cases. After this initial 10 ms, even though the light stimulus remains present and the membrane is relaxed, we hypothesized that the *trans-cis* isomerization induces some conformational changes in the membrane, allowing a small number of SACs to be opened. Once the stimulation ends, Ziapin2 dimers (i.e., *trans* configuration) are restored, enabling an increasing number of SACs to be activated. In fact, in the second phase ($t_{2}$), the duration of which varies depending on when the AP is triggered, a slow rise occurs with a small stretch applied. As more molecules undergo *trans-cis* isomerization, the stretch increases, reaching its peak in the third phase ($t_{3}$). Finally, in the fourth phase ($t_{4}$), the system returns to the equilibrium condition.

## Results

### Effects of membrane capacitance variations

**Figure 3:**
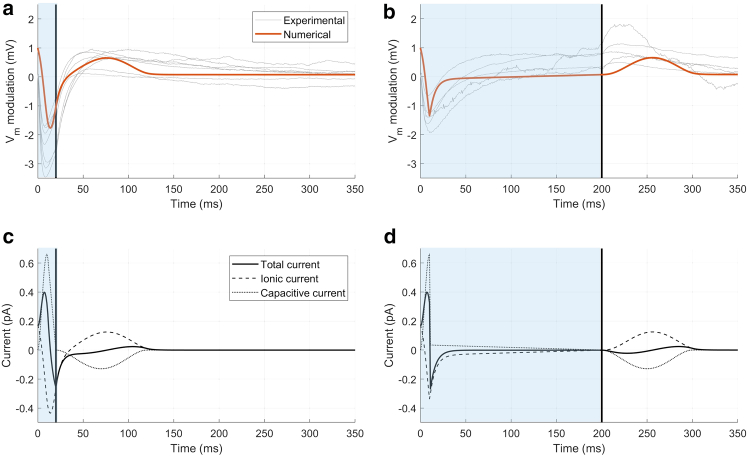
Membrane capacitance variation effects. (*a* and *b*) Comparison between representative experimental traces (23) and numerical simulations of the transmembrane potential simulating Gd^3+^ administration for two different light stimulation durations: 20 ms and 200 ms. (*c* and *d*) The corresponding total current, calculated as the sum of ionic and capacitive currents. Photoexcitation is represented by the blue shaded area, and the black line indicates the end of the light stimulation. $V_{m}$ values are reported as relative variation to emphasize the effects induced by light stimulation.

Two different durations of light stimulations were used to numerically reproduce experimental data (23) (i.e., 20 and 200 ms). Numerical simulations were performed using a single pulse, consistent with the experimental recordings. Additionally, the model was tested under repeated light stimulation at a frequency of 0.5 Hz (results not shown). In vitro, Ziapin2-loaded AMVMs exhibited a biphasic modulation of the membrane potential, characterized by an initial hyperpolarization coincident with the capacitance change, followed by a delayed depolarization. The hyperpolarization peak occurred with similar latency for both 20 and 200 ms stimuli. However, the peak depolarization was delayed with 200 ms stimuli, as shown in Fig. 3, *a* and *b*.

In the first phase of the study, we numerically replicated experimental data obtained from AMVMs treated with the SAC blocker gadolinium (Gd^3+^) (23) to disentangle the $V_{m}$ modulation induced by changes in $C_{m}$. Representative experimental traces after Gd^3+^ administration are shown in Fig. 3, *a* and *b*. Under these conditions, the magnitude and timing of hyperpolarization remained unaffected, while the amplitude of depolarization significantly decreased, preventing AP triggering. This suggests that, in the presence of Ziapin2, SAC blockade selectively affects depolarization mechanisms without altering hyperpolarization dynamics under light stimulation. Specifically, a hyperpolarization peak of −2.27 ± 0.2 mV and −1.63 ± 0.17 mV for 20 and 200 ms of light stimulation, respectively, was recorded, occurring at 11.64 ± 0.89 ms and 12.68 ± 1 ms after stimulation onset. The small depolarization had an amplitude of 1.53 ± 0.27 mV and 1.25 ± 0.25 mV for 20 and 200 ms of light stimulation, occurring at 72.9 ± 6.11 ms and 192 ± 15.3 ms, respectively (Fig. S2) (23).

The results for the two stimulations at 20 and 200 ms compared with experimental data are shown in Fig. 3, *a* and *b*. We see that the sole effect of the capacitance change, induced by the presence of Ziapin2 in the membrane, results in a hyperpolarization peak of −1.9 mV at 13 ms for the 20 ms light stimulation and −1.55 mV at 10 ms for the 200 ms stimulation. These peaks are followed by a small depolarization of 0.57 mV for both the 20 and 200 ms stimulations, occurring, respectively, at 74 and 254 ms (Fig. S2). It is worth noting that, under these conditions, the $V_{m}$ modulation is given by the sole effect of the extra term, resulting in a shape comparable with the one of $\frac{dC_{m}}{dt}$ (Fig. 1 *b*). In Fig. 3, *c* and *d*, the total current and the contributions of the ionic and capacitive currents are also shown. Numerical data highlight the role of the capacitive current as the main driving force in the behavior of the transmembrane potential during the initial phase of the dark-to-light transition. As expected for such subthreshold perturbations of membrane potential, sarcolemmal ionic currents are shown to largely compensate for the effect of the capacitive current as they work to bring the system to equilibrium (reestablishing the resting membrane potential).

### Effects of SAC presence

#### Contribution of individual SACs on the AP dynamics

**Figure 4:**
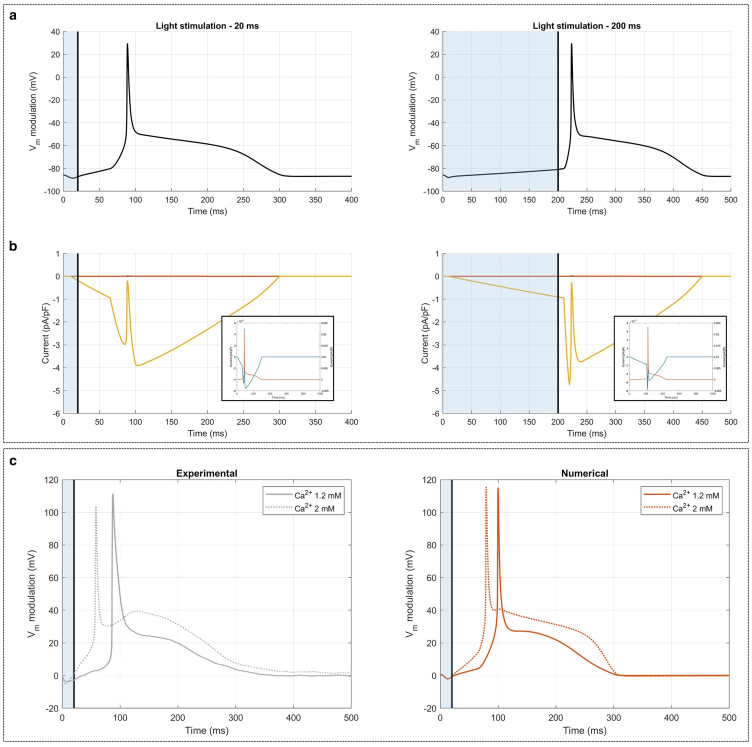
Numerical APs and their underlying currents, and extracellular Ca^2+^ effect of the mouse ventricular myocyte model. (*a*) Light-stimulated APs with stimulation duration of 20 ms (*left*) and 200 ms (*right*), respectively. The dotted line illustrates that the inactivation of Ca^2+^-selective SACs prevents AP generation. (*b*) SACs currents underlying the light stimulated AP. (*c*) Experimental (23) (*left*) and numerical (*right*) transmembrane potential simulated with 1.2 and 2.0 mM of extracellular Ca^2+^ concentration for 20 ms light stimulation duration. Photoexcitation is represented by the blue shaded area, and the black line indicates the end of the light stimulation. $V_{m}$ values are reported as relative variations to emphasize the effects induced by light stimulation.

By incorporating SACs into the numerical model and adding their contribution to the effect of variations in membrane capacitance, we successfully replicated the experimentally observed AP triggering (Fig. 4). Specifically, the transmembrane potential exhibited an initial small hyperpolarization peak, similar to that observed after Gd^3+^ administration (23) (i.e., in numerical simulations where action of SACs was neglected) and attributable to variations in membrane capacitance. This was followed by a gradual depolarization during light stimulation, culminating in AP triggering once the light stimulation was interrupted.

Various scenarios were considered in silico by selectively blocking SACs. The results show that blocking nonselective SACs or K^+^-selective SACs still allows the threshold for AP generation to be reached (results not shown), whereas blocking Ca^2+^-selective SACs prevents AP generation (*dotted lines* in Fig. 4 *a*). This is particularly evident when examining individual SACs currents (see Fig. 4 *b*). The figure shows that the currents associated with nonselective and K^+^-selective SACs are much smaller compared with the Ca^2+^-selective SACs. These tests revealed that only the Ca^2+^-selective SACs contributed to AP triggering, corroborating the educated hypothesis derived experimentally (23).

To further support the important role of Ca^2+^-selective SACs, we numerically reproduced experimental analyses with different extracellular Ca^2+^ concentrations, using 2 mM as the control and 1.2 mM as the reduced concentration. Examining the experimental traces shown in Fig. S2, two distinct AP shapes can be observed for both 20 and 200 ms light stimulations: one more triangular (Fig. S2, *a* and *d*), resembling the typical murine AP shape generated by electrical stimulation, and the other with a more prolonged plateau phase (Fig. S3, *b* and *e*). This variation in AP shape depends on the extracellular Ca^2+^ concentration used. Specifically, for the control condition (i.e., 2 mM), none of the experimental traces exhibit a triangular shape; instead, all show a significantly prolonged plateau phase (Fig. S3, *c* and *f*). In contrast, reducing the extracellular Ca^2+^ concentration to 1.2 mM results in APs with gentler slopes and less prolonged plateau phase (Fig. S3, *a*, *b*, *d*, and *e*). This observation was confirmed numerically, as shown in Fig. 4 *c*. The left panel shows illustrative experimental recordings for the two Ca^2+^ concentrations, and the right panel shows numerical simulations using the same two Ca^2+^ concentrations with a 20 ms light stimulation. In both cases, reducing the extracellular Ca^2+^ concentration to 1.2 mM led to APs with lower upstroke velocity compared with the control condition with 2 mM, and shorter AP duration (APD). In addition, numerically simulating a near-zero extracellular Ca^2+^ concentration prevented the generation of an AP (result not shown).

A significant feature observed in experimental recordings on AMVMs is the prolongation of APs evoked by light stimulation compared with those generated by electrical stimulation. While electrically stimulated APs typically exhibit a triangular shape with an APD at 90% repolarization (APD_90_) of approximately 50–100 ms, light stimulation increases this value more than fivefold, with this increase being dependent on the length of the light pulse (23). For light pulses between 20 and 200 ms, the average APD_90_ increases from 287.4 ± 19.6 ms to 402 ± 19 ms (a 39.8% increase) (23). This effect was also evident numerically, where APD_90_ increased from 13.09 ms (for electrical stimulation) to an average of 248.78 ± 14.51 ms and 368.75 ± 18.18 ms for light stimulation durations of 20 and 200 ms, respectively (Fig. S4), which implies a more than fivefold increase in APD_90_ with respect to electrical stimulation, and an increase of 21.73% in APD_90_ between light pulses between 20 and 200 ms.

**Figure 5:**
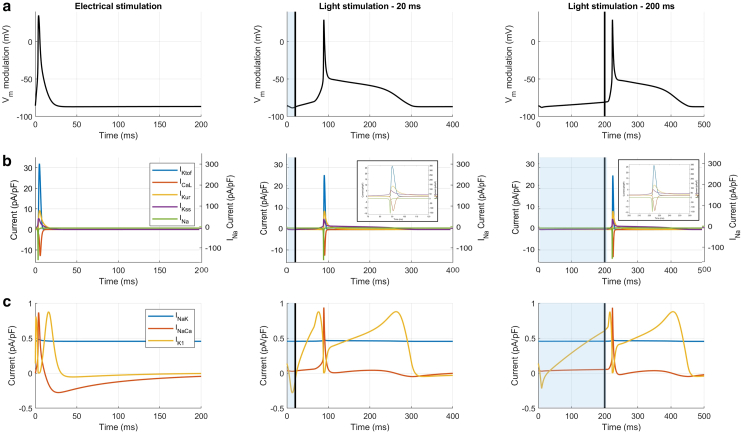
Comparison between electrical and light-stimulated AMVCMs. (*a*) Numerical APs and (*b*) and (*c*) underlying currents of the AMVCMs model for the electrical and light stimulated (20 and 200 ms) AP. Photoexcitation is represented by the blue shaded area and the black line indicates the end of the light stimulation. $V_{m}$ values are reported as relative variation to emphasize the effects induced by light stimulation. The scale for the relatively large Na^+^ current $I_{Na}$ is given on the right axis in (*b*). All other currents are scaled on the left axis.

To better understand the underlying mechanisms, we analyzed the currents activated during light-induced APs, comparing them to those induced by traditional electrical stimulation (Fig. 5 *a*). From the comparison in Fig. 5 *b*, no significant differences emerge, except for a small reduction in $I_{Ktof}$, $I_{Na}$, and $I_{CaL}$ during light-induced APs. In Fig. 5 *c*, however, a clear difference in $I_{K1}$ is observed. During electrical stimulation, $I_{K1}$ has a brief duration consistent with the triangular AP shape and shorter APD. In contrast, during light-evoked APs, $I_{K1}$ is prolonged, supporting the extended plateau phase. This prolonged plateau is sustained by the activity of SACs, particularly by the Ca^2+^-selective ones, as shown in Fig. 4, *c* and *d*.

In relation to $I_{Na}$, our numerical simulations show that the Na^+^ current amplitude during the triggered upstroke decreases as the stimulus duration is prolonged (Fig. S5). This results in longer subthreshold depolarizations, indicating that Na^+^ channels partially inactivate during the subthreshold phase of optical stimulation. However, successful AP triggering is still observed, implying that enough Na^+^ channels remain available to support the AP upstroke. Notably, the activation threshold remains similar between the two pulse durations, suggesting that the reduction in I_Na_ availability is inconsequential for triggering the AP.

Equally surprisingly, the Na^+^-Ca^2+^ exchanger (NCX) does not seem to have a significant contribution in the process. Indeed, even when an influx of extra Ca^2+^ occurs during light stimulation, the resulting higher intracellular concentration does not appear to significantly affect the NCX current. This limited role can be attributed to the plateau potential of the AP, which is relatively close to the NCX reversal potential. As a result, the driving force for the NCX current is reduced, thereby limiting its contribution during the AP (see Fig. S6). Moreover, by analyzing Ca^2+^ flux uptake by the SERCA pump and the sarcoplasmic reticulum Ca^2+^ concentration (Fig. S7), it was evident that the additional influx of Ca^2+^ is stored within the sarcoplasmic reticulum during light stimulation.

#### Kinetics of AP triggering

Analyzing the experimental traces (Fig. S3) the high variability in the timing of AP triggering among the traces was evident, especially for the 20 ms light stimulation duration. Notably, the AP peak occurred at 146.09 ± 15.31 ms (*n* = 32 variability coefficient equals to 59.26%) after the stimulation ended for a 20 ms light stimulation and 42.25 ± 10.39 ms (*n* = 52 variability coefficient equals to 177.3%) for a 200 ms light stimulation. Moreover, in some cases with 200 ms light stimulation, it is evident that $V_{m}$ can reach the threshold before the termination of the optical stimulation.

**Figure 6:**
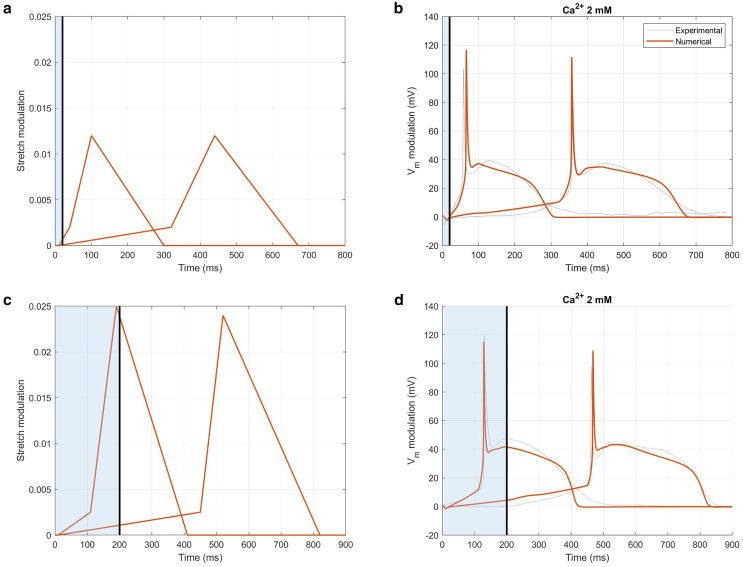
Numerical fitting of the experimental extreme cases for 2 mM Ca^2+^ extracellular concentration. (*a* and *c*) Stretch variations applied to fit extreme cases and (*b* and *d*) and their corresponding transmembrane potential for 20 and 200 ms light stimulation. Photoexcitation is represented by the blue shaded area, and the black line indicates the end of the light stimulation. $V_{m}$ values are reported as a relative variation to emphasize the effects induced by light stimulation.

To numerically reproduce this variability and better understand the molecule functioning, different amplitudes and stretch timing were numerically assessed to fit the extreme cases, as reported in Fig. 6. In particular, for both light stimulation durations, the cases in which the AP was generated with a shorter and a longer timing were selected.

During the first 10 ms (corresponding to the hyperpolarization time to peak), SACs are not yet activated and, in fact, no stretch is applied. A slight stretch is necessary to establish the initial slope, with extreme cases showing a 0.2–0.25% of stretch. This phase is associated with the *trans-cis* isomerization of the molecule induced by the light stimulation. Following this, a more significant stretch, ranging from 1.2 to 2.5% in 20 and 200 ms of light stimulation, respectively, is required to trigger the AP. This phase corresponds to the molecule’s dimer restoration associated with the return to the dark condition. Note that these values are quite close to the 3% stretch of the cell membrane due to the changes in cell membrane thickness during light stimulation. In Fig. S8 the fittings of extreme cases for a reduction to 1.2 mM of Ca^2+^ extracellular concentration are also reported. From this analysis, it was evident that the amount of stretch applied—and thus the number of activated SACs—was similar across extreme cases. Instead, to reproduce the experimentally observed variability in the triggering of the APs, it was necessary to adjust the timing of the four phases of stretch modulation, as shown in Fig. 6. This timing difference is particularly evident in $t_{2}$, which corresponds to the moment when the AP is triggered.

**Figure 7:**
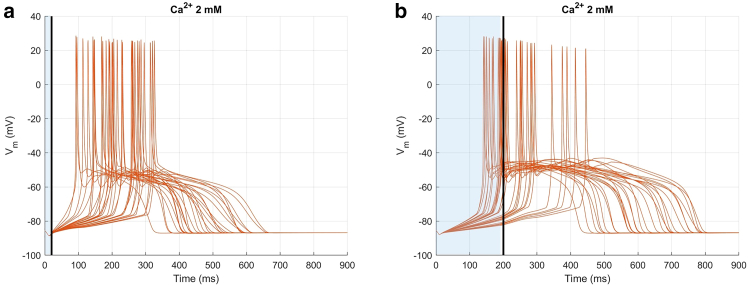
Numerical AP variability. Numerical transmembrane potentials for two different light stimulation durations. (*a*) Twenty millisecond light stimulation and (*b*) 200 ms light stimulation. Photoexcitation is represented by the blue shaded area, and the black line indicates the end of the light stimulation. $V_{m}$ values are reported as relative variation to emphasize the effects induced by light stimulation.

Once the extreme cases were numerically fitted, different numerical cases with random values (i.e., $t_{1}$, $t_{2}$, $t_{3}$) within the selected experimental ranges were then simulated, with the extreme cases representing the boundary values of this range, to reproduce the variability observed experimentally. Particularly, for 20 ms light stimulation $t_{1}$ was 10 ms in both extreme cases, while $t_{2}$ was 40 and 320 ms, $t_{3}$ was 100 and 440 ms, and $t_{4}$ was 300 and 670 ms, respectively. A stretch modulation of 0.2% was applied in the first phase, followed by 1.2% in the second phase, for both extreme cases. Similarly, for 200 ms of light stimulation, $t_{1}$ was 10 ms in both extreme cases, while $t_{2}$ was 110 and 450 ms, $t_{3}$ was 190 and 520 ms, and $t_{4}$ was 410 and 820 ms, respectively. A stretch modulation of 0.25% was applied in the first phase, followed by 2.5 and 2.4% in the second phase, for the lower and upper extreme cases, respectively. This suggests that, to generate the experimentally observed variability, the activation timing of the stretch must be modified while keeping its intensity unchanged. The result is shown in Fig. 7 and Table 3, while in Fig. S9 the sensitivity analysis for a reduction to 1.2 mM of Ca^2+^ extracellular concentration is also reported.

**Table 3:** AP features

Parameter	20 ms	200 ms
${dV/dt}_{\max1}$ (mV/ms)	0.23 ± 0.03	0.12 ± 0.02
Threshold of AP activation (mV)	−76.78 ± 0.51	−74.23 ± 1.58
${dV/dt}_{\max2}$ (mV/ms)	82.61 ± 7.49	83.34 ± 10.54
${APD}_{90}$ (ms)	224.90 ± 71.70	348.10 ± 94.82
RMP (mV)	−86.76	−86.74
Peak $I_{Na}$ (pA/pF)	112.35 ± 18.88	101.10 ± 17.93

Numerical AP features obtained from the Monte Carlo simulation. *N* = 30.

**Figure 8:**
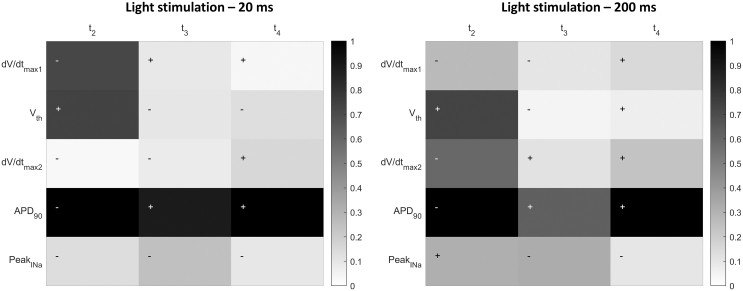
Partial correlation. Numerical partial correlation between AP features and stretch activation timing for two different light stimulation durations: 20 ms light stimulation (*left*) and 200 ms light stimulation (*right*).

Moreover, a partial correlation analysis was conducted to identify which timing parameter of the stretch most influences the AP features and to determine which specific aspects are more or less affected. The results for both 20 and 200 ms light stimulation are reported in Fig. 8. The analysis reveals that the most impacted parameter is APD_90_, while the temporal parameter that has the greatest influence is $t_{2}$, as it determines the onset of a more intense stretch ramp. This, in fact, is responsible for the variations in the slow subthreshold depolarization slope (${dV/dt}_{\max\;1}$) and the threshold of activation ($V_{th}$) (although Table 3 shows that this parameter exhibits minimal variation across different cases). APD_90_ is influenced by all timing parameters, as they collectively determine the various phases of the AP. Finally, the peak of the Na^+^ current (peak $I_{Na}$) is primarily influenced by the parameter $t_{3}$, as it governs the point of maximum stretch and is thus associated with the generation of the AP.

## Discussion

In this study, we modified a computational model of murine AP (26) to investigate the effects of Ziapin2, a newly synthetized light-sensitive azobenzene compound (16,19,20,21,22,32), on cardiac cellular electrophysiology. Experimentally, we observed that, upon light stimulation, cells containing Ziapin2, present an initial membrane hyperpolarization, followed by a delayed depolarization. The hyperpolarization has been attributed to changes in membrane thickness, which in turn affect cell membrane capacitance. This phenomenon occurs in both excitable (16,22) and nonexcitable cells (17,18). In nonexcitable cells, the depolarization is minimal, consistent with capacitance recovery. In contrast, in cardiac excitable cells, the depolarization is significantly larger, exceeding the threshold needed for AP generation, suggesting the involvement of specific cellular mechanisms. The origin of the large depolarization, in terms of involved ion channels, transmembrane currents, and their timescales, is the main object of this work.

Experiments conducted in AMVMs have demonstrated, through pharmacological blockade, that cell depolarization occurring during light stimulation is associated with the activation of nonselective SACs. Further, the experimental findings support the hypothesis that Ca^2+^ influx through SAC_ns_ might be crucial to the whole process (23). These observations have motivated the incorporation of nonselective SACs in the murine model to further investigate the underlying mechanisms of AP generation under light stimulation on cells containing Ziapin2.

The model was validated by comparing its results with experimental data acquired in AMVMs (23). First, the model explains the lack of resting membrane potential change under dark conditions (in the presence of the molecule) that was observed experimentally (23). This supports the evidence that Ziapin2 does not affect the main transporters and receptors located within the sarcolemma. In addition, the numerical results confirmed that the model accurately and consistently replicated the observed data for light stimulation, both with and without inhibition of SACs, using Gd^3+^. Specifically, when the model considers only the contribution of $C_{m}$, it aligns with experimental data, showing an initial hyperpolarization peak followed by a small and delayed depolarization. The hyperpolarization peak remains consistent with varying durations of light stimulation, indicating that the initial segment of $C_{m}$ formulation remains invariant ($t_{1}$ is consistently 10 ms). The return to the resting value is also similar for both stimulations. The main experimental difference between 20 and 200 ms light stimulation is the onset time of depolarization. In the numerical model, this is adjusted by setting the vertex of the parabola (the third segment) to correspond with the duration of light stimulation ($t_{3}$). Depolarization occurs after stimulation ends, and the "rebound" has similar duration and amplitude for both light stimulations, suggesting that the interval $t_{3}$-$t_{4}$ of the fourth segment is consistent but shifted in its starting point.

Both experimental and numerical results showed the same hyperpolarization even without SAC activity, indicating that this hyperpolarization can be entirely attributed to variations in $C_{m}$. When considering SAC involvement, APs can be triggered. A gradual depolarization follows the initial hyperpolarization peak in transmembrane potential, linked to channel stretching caused by changes in membrane thickness introduced by Ziapin2 during light stimulation. To achieve this gradual depolarization, a degree of stretch is necessary during this phase, which can last beyond the light stimulation. This phase significantly contributes to the variability in AP triggering timing. Analysis of extreme cases shows that, while the extent of stretch modulation remains consistent across cases—suggesting a similar number of molecules inserted into the membrane, thus generating a comparable SACs activity—the timing of stretch modulation, particularly during $t_{2}$, varies significantly. This high variability in AP triggering timing after the termination of light stimulation could stem from a distribution of dimerization rates, or perhaps the formation of multimers. Studies on the isomerization dynamics of Ziapin2 in different media (17) report time constant on the order of picoseconds to nanoseconds. However, since no data are available on the collective dynamics within the membrane, this possibility cannot be excluded. On the other hand, differences in SAC opening kinetics or the membrane mechanical response (e.g., viscosity) to Ziapin2 isomerization could also be hypothesized. For the former, experimental evidence has demonstrated that SAC opening occurs rapidly (in the range of 10–100 ms), with more variability in the closing process (33,34,35). However, this variability cannot explain the large differences in AP triggering timing. In experiments performed using colchicine (23) to disrupt the cytoskeleton, the light stimulus still resulted in AP triggering. This suggests that cytoskeletal properties are unlikely to account for the observed variability in AP onset.

## Conclusions

Overall, the numerical model accurately reproduces the changes in cell capacitance and membrane potential induced by Ziapin2 photoisomerization, clarifying the experimentally observed behavior AMVMs. The model confirms the crucial role of SACs in AP generation, with a particular emphasis on the involvement of Ca^2+^-selective channels, and also enables exploration of aspects that could not be investigated experimentally, as is the Ca^2+^ accumulation in the sarcoplasmic reticulum after repeated light stimulation. In summary, the model suggests that, in “dark” conditions, the cell finds a new homeostatic state that does not alter its basic electrophysiological characteristics at rest or when electrically stimulated. Upon entering the cell membrane, the molecule induces conformational changes that alter membrane thickness and surface area. Supported by various conductances balancing ionic fluxes, AMVMs establish a new homeostatic state. During light stimulation, the molecule *trans-cis* isomerization restores the membrane thickness, causing the channel closing. In this phase, a slight stretch affects a small number of SACs due to membrane irregularities. After light stimulation stops, the molecule dimerizes, returning to the *trans* configuration, which increases membrane stretch and allows Ca^2+^ to flow into the cell through open Ca^2+^-selective channels. Eventually, the membrane returns to its equilibrium state.

The availability of this in silico model, complementing experimental findings, provides a comprehensive understanding of the Ziapin2-mediated photostimulation process and it is a validated tool for predicting Ziapin2 effects before experiments. Overall, the acquired knowledge about this new photostimulation process opens promising prospects for its application in cardiovascular research.

## Data and code availability

The CellML model along with the MATLAB code are available on GitHub (https://github.com/LudovicaCestariolo/Ziapin2), and the data that support the findings of this study are available from the corresponding authors upon reasonable request.

## Acknowledgments

This study was supported by the 10.13039/501100003407Italian Ministry of Education, University and Research through the PRIN 2022 project (ID 2022-NAZ-0595) awarded to F.L. and supporting L.C., the PRIN 2020 project (ID 2020XBFEMS) awarded to C.B. and G.L., the Fondo Italiano per la Scienza project (ID FIS00001244) awarded to G.L., and a grant from the 10.13039/501100003407Italian Ministry for Education, University and Research (grant no. 1613 FISR2019_03221, CECOMES) awarded to J.F.R.M.

## Author contributions

Conceptualization, L.C., F.L., and J.F.R.M.; data curation, L.C.; formal analysis, L.C.; investigation, L.C.; methodology, L.C. and J.F.R.M.; software, L.C. and J.F.R.M.; visualization, L.C.; writing – original draft, L.C., F.L., and J.F.R.M.; writing – review & editing, L.C., C.F., C.B., A.Z., G.L., F.L., and J.F.R.M.; experimental data acquisition and curation, C.F.; funding acquisition, C.B., G.L., F.L., and J.F.R.M.; resources, C.B.; experimental data acquisition supervision, F.L.; project administration, F.L. and J.F.R.M.

## Declaration of interests

C.B., G.L., and F.L. are inventors of “PHOTOCHROMIC COMPOUNDS" patent no. EP 3802491 (02/07/2020).

## Supporting material

Document S1. Figures S1–S9 and Table S1

Document S2. Article plus supporting material
