# Supplementary material for: Cardiac action potential generation mechanisms via an intramembrane photoswitch. A simulation study
Source: Biophys J. 2025 May 3;124(24):4505–16. doi: 10.1016/j.bpj.2025.04.029 (PMC12820999; doi:10.1016/j.bpj.2025.04.029)
Supplement: Document S1. Figures S1–S9 and Table S1 [file mmc1.pdf]

Biophysical Journal, Volume *124*

Supplemental information

Cardiac action potential generation mechanisms via an intramem-brane photoswitch. A simulation study

Ludovica Cestariolo, Chiara Florindi, Chiara Bertarelli, Antonio Zaza, Guglielmo Lanzani, Francesco Lodola, and Jose F. Rodriguez Matas

Supplemental Information

Modellization of C_m variations over time

The first segment (from t_0 to t_1) is characterized by a Boltzmann function, represented by Eq. S1, whose derivative (Eq. S2) reflects the descending phase of the transient hyperpolarization observed experimentally in the presence of the molecule.

$$C_{m1} = 1.14 + (A_{max} - 1) \cdot \xi_1^2 \cdot (a - b\xi_1 + c\xi_1^2) \quad \text{(S1)}$$

$$\frac{dC_{m1}}{dt} = (A_{max} - 1) \cdot \xi_1^2 \cdot \frac{(3a - 4b\xi_1 + 5c\xi_1^2)}{2t_1 - t_0} \quad \text{(S2)}$$

with $\xi_1 = \frac{t - t_0}{2t_1 - t_0}$. Consequently, the V_{50} of the Boltzmann corresponds to t_1 and a C_m value intermediate between dark and light values. This point will correspond to the minimum value in the derivative, generating a hyperpolarization peak in the transmembrane potential due to the presence of the extra term in the parallel conductor model.

The second and third segments (from t_1 to t_2 and from t_2 to t_3) are characterized by a connection ensuring continuity in the derivative and a parabola with its vertex in t_3 corresponding to the minimum value of C_m (i.e., under light conditions). The time at which this value is reached will be determined by the duration of the imposed light stimulation. The equations for C_m and its derivatives in segments 2 and 3 are expressed as follows:

$$C_{m2} = dt^3 + et^2 + ft + g \quad \text{(S3)}$$

$$\frac{dC_{m2}}{dt} = 3dt^2 + 2et + f \quad \text{(S4)}$$

$$C_{m3} = ht^2 + it + l \quad \text{(S5)}$$

$$\frac{dC_{m3}}{dt} = 2ht + i \quad \text{(S6)}$$

Lastly, for the fourth segment (from t_3 to t_4) a Boltzmann equation was used represented by Eq. S7, whose derivative (Eq. S8) reflects the delayed depolarization observed experimentally in the presence of the molecule.

$$C_{m2} = A_{max} + 0.14 + (1 - A_{max}) \cdot \xi_2^3 \cdot (a - b\xi_2 + c\xi_2^2) \quad \text{(S7)}$$

$$\frac{dC_{m2}}{dt} = (1 - A_{max}) \cdot \xi_2^2 \cdot \frac{(3a - 4b\xi_2 + 5c\xi_2^2)}{t_4 - t_3} \quad \text{(S8)}$$

with $\xi_2 = \frac{t - t_3}{t_4 - t_3}$.

Table S1. C_m parameters. *Constants variable used for C_m formulation in case of 20 ms and 200 ms of light stimulation.*

Parameter	20 ms l.s.	200 ms l.s.
a	10	10
b	15	15
c	6	6
d	1.562499999999929e-04	-0.002071963028169
e	-0.0048046875	0.068375692063565
f	0.042656249999997	-0.752487432820605
g	1.002656250000009	3.864268150018524
h	3.515624999999990e-04	9.121339881393804e-07
i	-0.0140625	-3.648535952557498e-04
l	1.210625	1.106485359525575
A_{max}	0.93	0.93

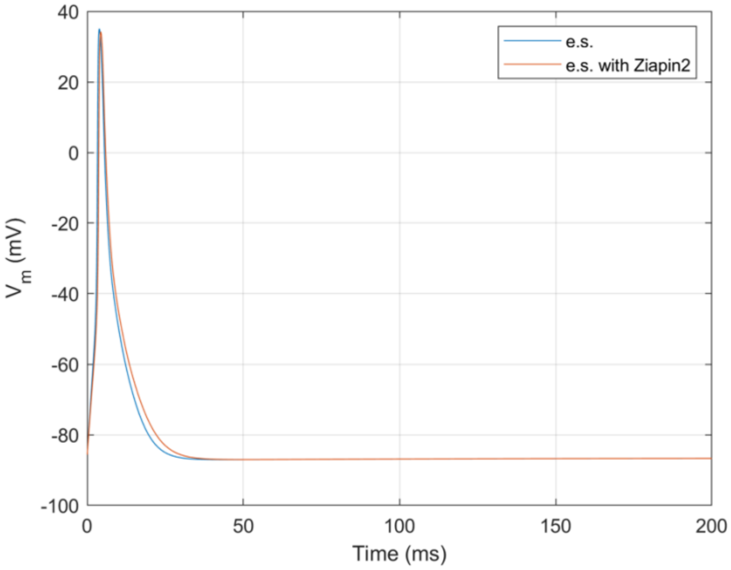

Figure S1. Numerical simulation of action potential dynamics under baseline conditions and with increased membrane capacitance (i.e., 1.14, representing the "dark" condition). *The figure shows that no significant differences were observed between the two conditions. The control/original model is represented in blue, while the "dark" condition is shown in orange.*

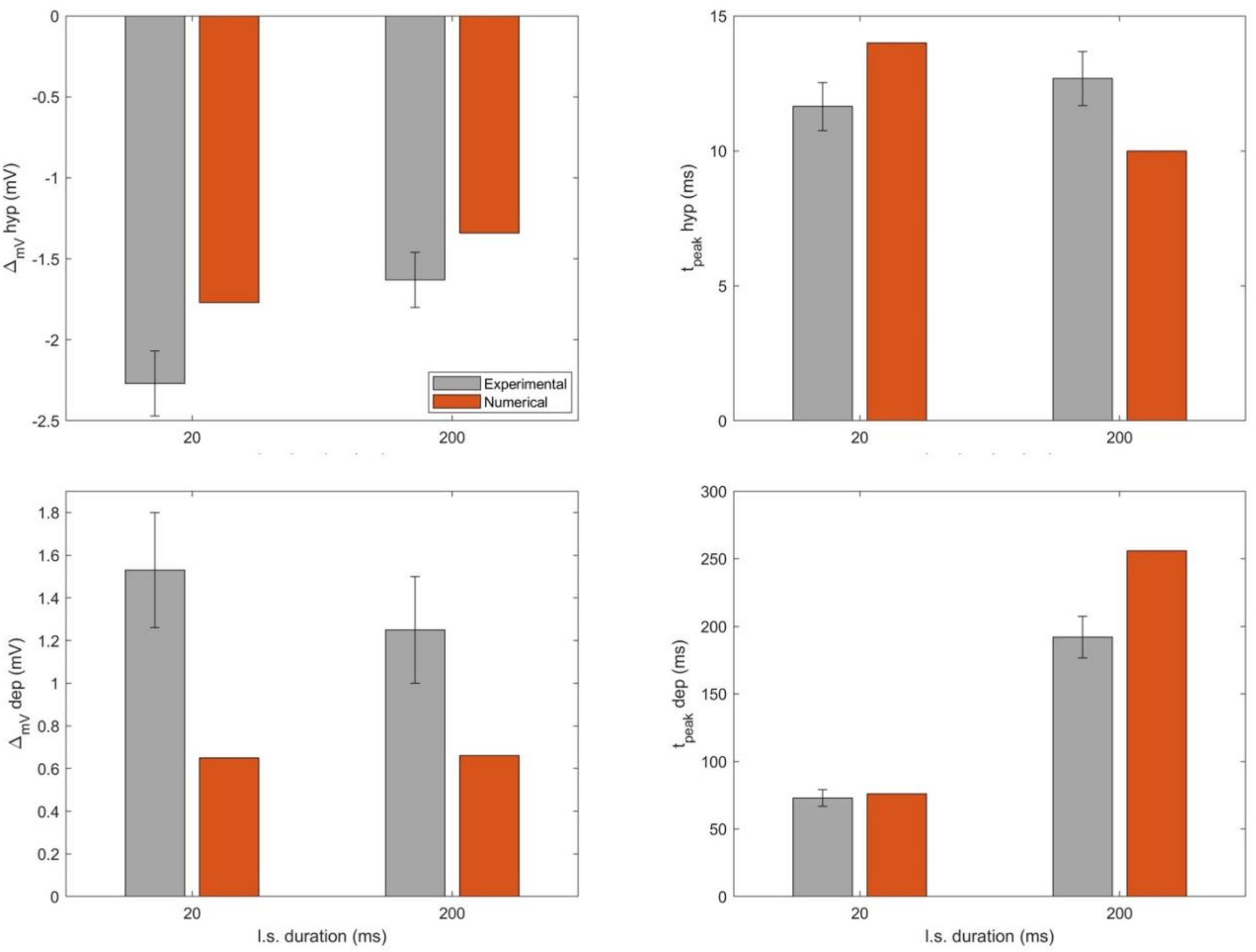

Figure S2. Membrane capacitance variation effects on hyperpolarization and depolarization peaks. *Comparison between experimental and numerical peak hyperpolarization and depolarization changes in Ziapin2-loaded AMVMs exposed to 25 µM Ziapin2. Experimental hyperpolarization peak 20 ms: −2.27 ± 0.2 mV; hyperpolarization peak 200 ms: −1.63 ± 0.17 mV; time to peak hyperpolarization 20 ms: 11.64 ± 0.89 ms; time to peak hyperpolarization 200 ms: 12.68 ± 1 ms; depolarization peak 20 ms: 1.53 ± 0.27 mV; depolarization peak 200 ms: 1.25 ± 0.25 mV; time to peak depolarization 20 ms: 72.9 ± 6.11 ms; time to peak depolarization 200 ms: 192 ± 15.3 ms. Numerical hyperpolarization peak 20 ms: -1.77 mV; hyperpolarization peak 200 ms: -1.34 mV; time to peak hyperpolarization 20 ms: 14 ms; time to peak hyperpolarization 200 ms: 10 ms; depolarization peak 20 ms: 0.65 mV; depolarization peak 200 ms: 0.66 mV; time to peak depolarization 20 ms: 76 ms; time to peak depolarization 200 ms: 256 ms. Data are represented as mean ± SEM.*

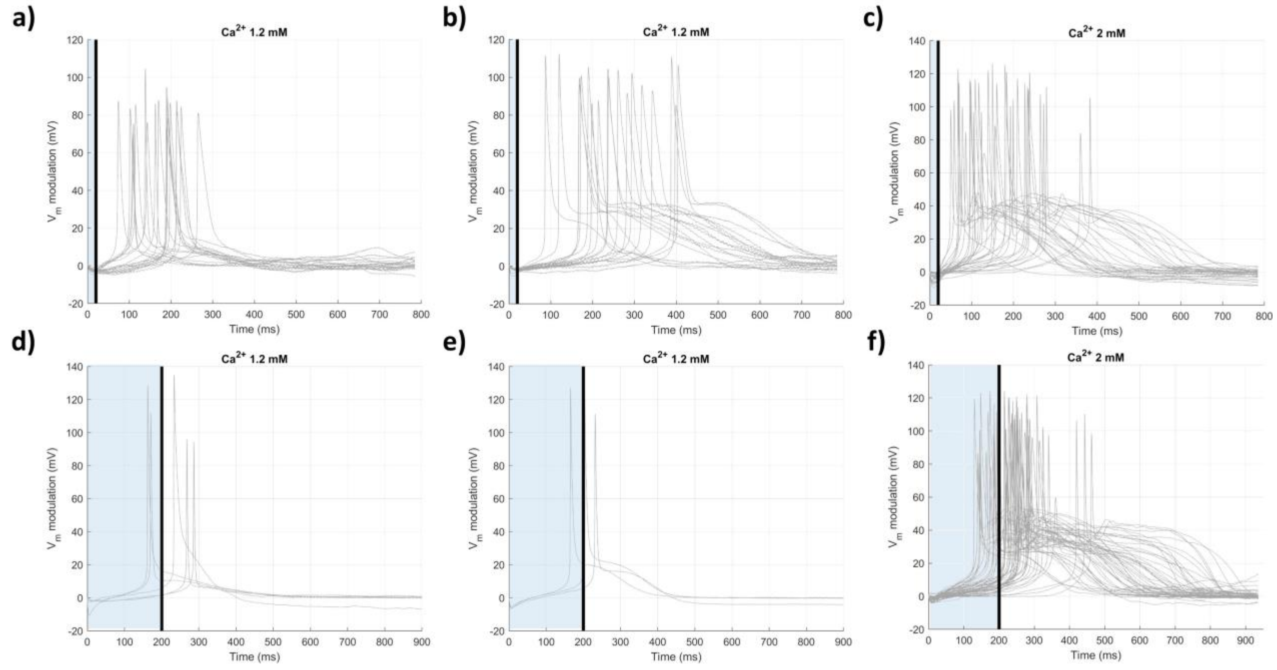

Figure S3. Experimental transmembrane potentials. *The results were obtained for two different light stimulation durations. Panel* ***a) – c)****: 20 ms light stimulation. Panel* ***d) – f)****: 200 ms light stimulation. Photoexcitation is represented by the blue shaded area, and the black line indicates the end of the light stimulation.* V_m *values are reported as relative variation to emphasize the effects induced by light stimulation.*

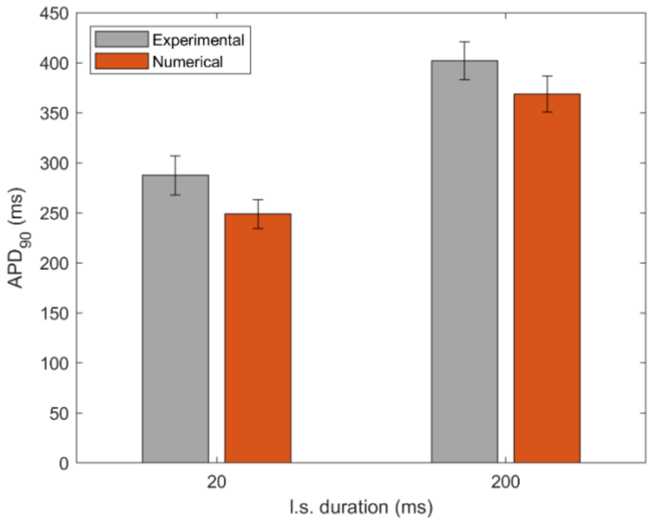

Figure S4. APD$_{90}$ during light stimulation. *Experimental and numerical APD$_{90}$ with extracellular Ca^{2+} equals to 2 mM for 20 ms and 200 ms light stimulation. Experimental APD$_{90}$ 20 ms: 287.4 ± 19.6, n = 30; APD$_{90}$ 200 ms: 402 ± 19 ms, n = 24. Numerical APD$_{90}$ 20 ms: 248.78 ± 14.51 ms, n = 30; APD$_{90}$ 200 ms: 368.75 ± 18.18, n = 30. Data are represented as mean ± SEM.*

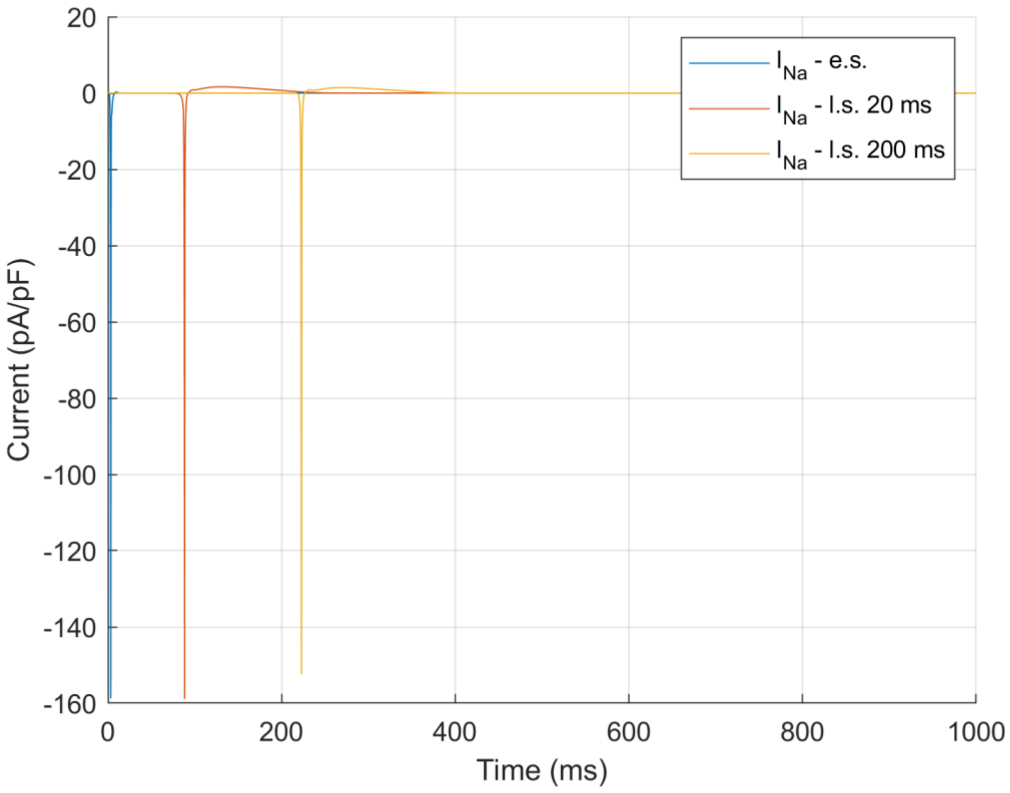

Figure S5: Sodium current (I_{Na}) during electrical (e.s.) and light-evoked (l.s.) APs. *Optical stimuli of 20 and 200 ms are shown.*

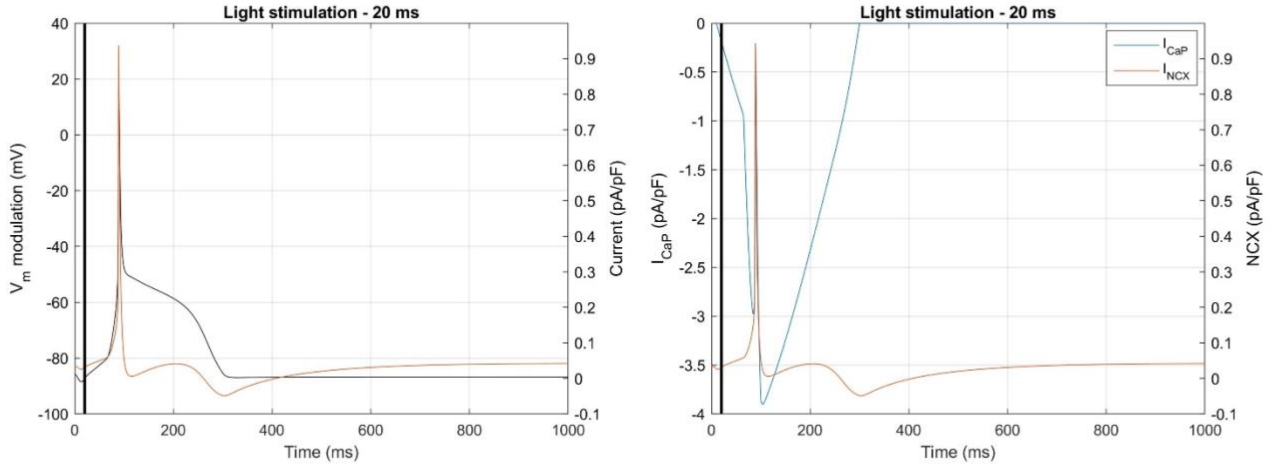

Figure S6: Contribution of NCX due to the plateau potential of the action potential*. a) The action potential waveform is shown in relation to the NCX reversal potential. b) NCX compared to the SAC_{Ca} current I_{CaP}. The black line indicates the end of the light stimulation.*

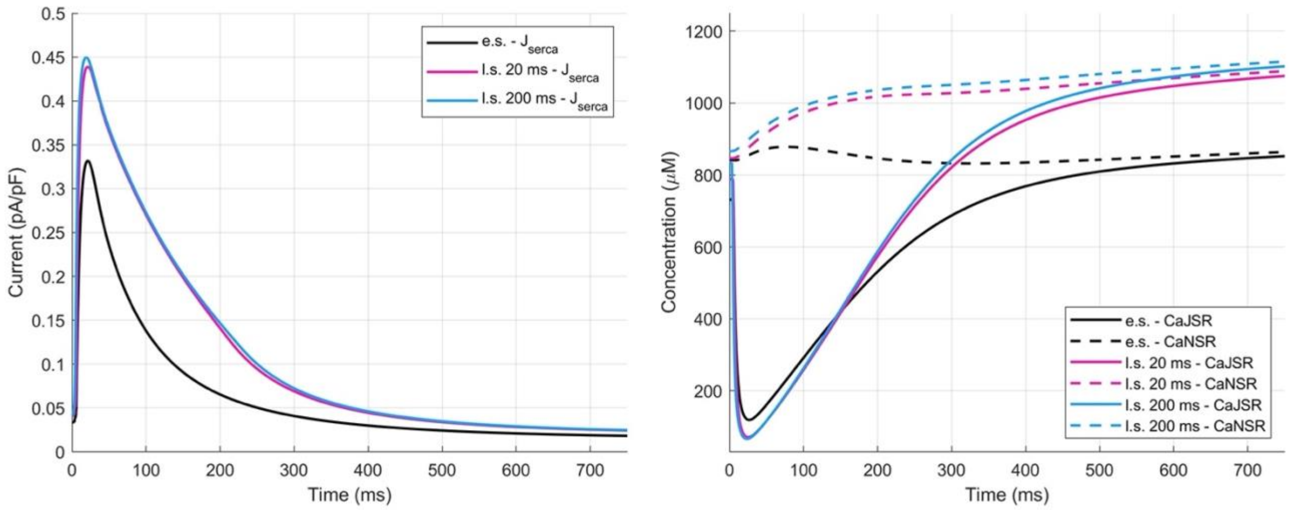

Figure S7. Comparison between electrical and light stimulated SR activity. *Variations in SERCA current (J_{SERCA}) (left) and Ca^{2+} concentrations in the junctional (CaJSR) and non-junctional (CaNSR) sarcoplasmic reticulum (right) during electrical and light stimulation. The figure highlights the increased calcium uptake observed during light stimulation.*

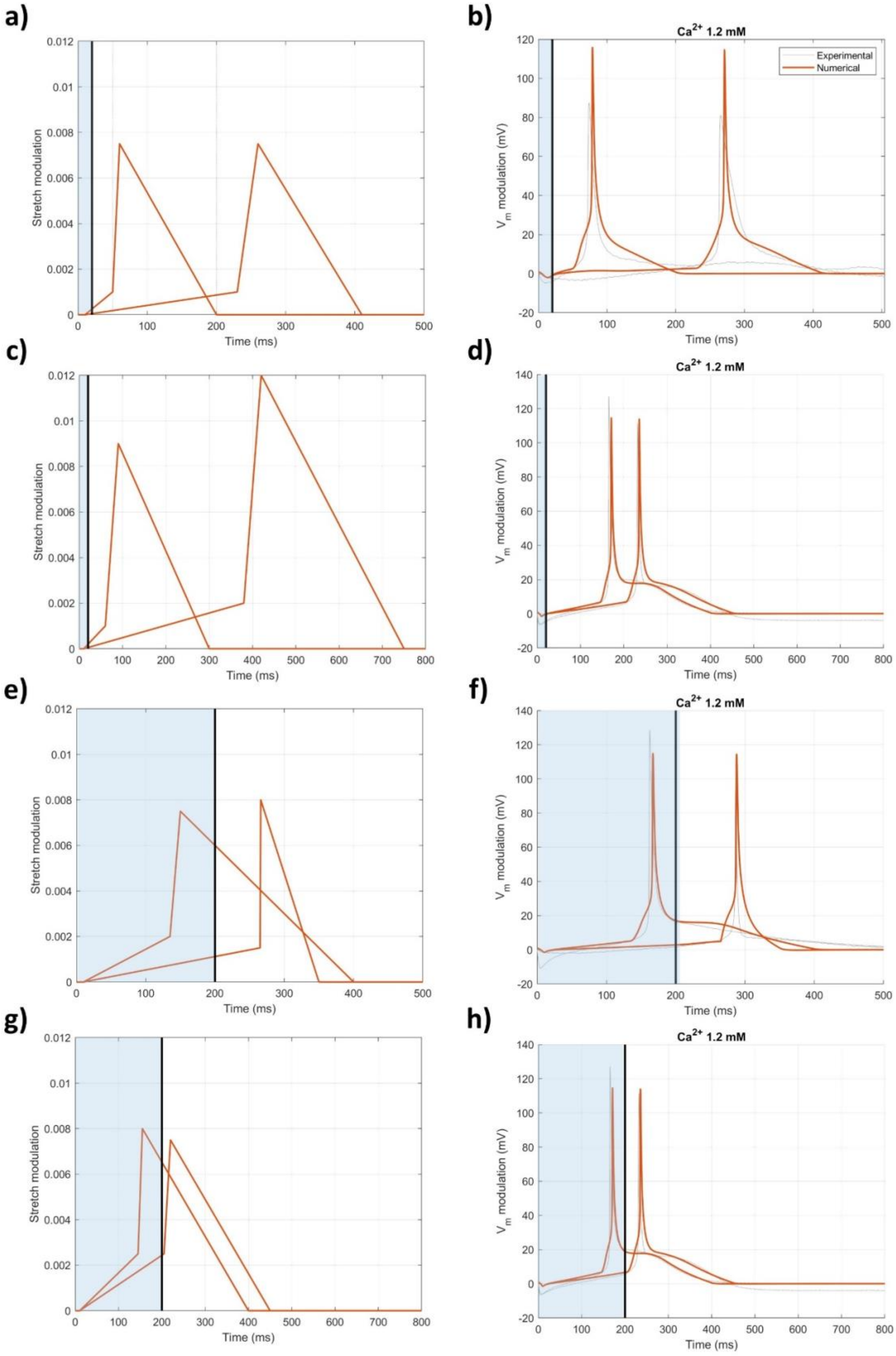

Figure S8. Numerical fitting of the experimental extreme cases for Ca^{2+} extracellular concentration reduced to 1.2mM. a) c) e) *stretch variations applied to fit extreme cases and* ***b) d) f)*** *and their corresponding transmembrane potential for 20 ms and 200 ms light stimulation. Photoexcitation is represented by the blue shaded area, and the black line indicates the end of the light stimulation.* V_m *values are reported as relative variation to emphasize the effects induced by light stimulation.*

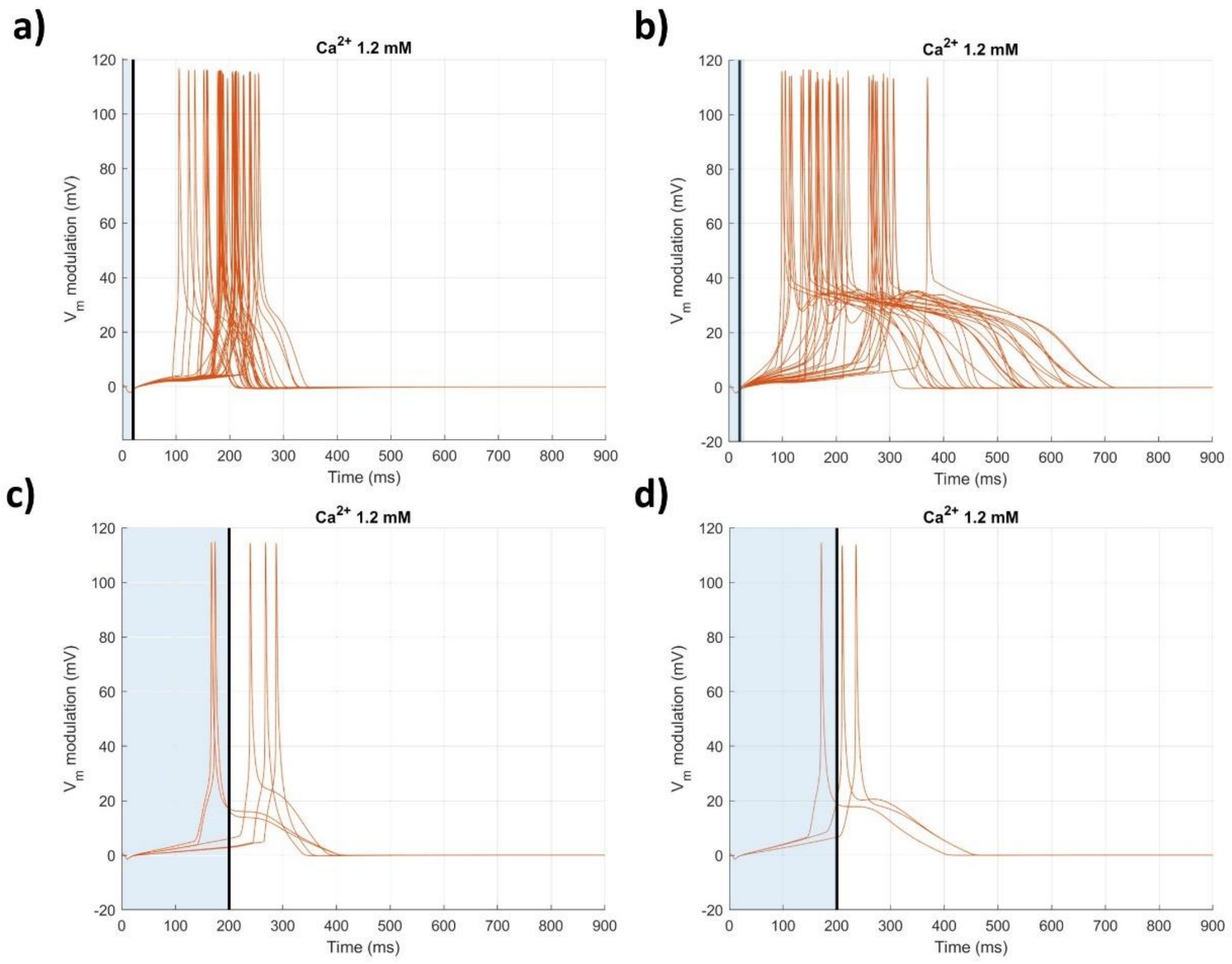

Figure S9. Numerical AP variability. *Numerical transmembrane potentials for two different light stimulation durations.* ***a – b)*** *20 ms light stimulation.* ***c – d)*** *200 ms light stimulation. Photoexcitation is represented by the blue shaded area, and the black line indicates the end of the light stimulation.* V_m *values are reported as relative variation to emphasize the effects induced by light stimulation.*
